# The role of age, grade level, and subject area in determining the inclusion of digital citizenship elements in elementary school curricula: Perspectives of teachers in the Kingdom of Saudi Arabia

**DOI:** 10.1016/j.heliyon.2024.e34597

**Published:** 2024-07-14

**Authors:** Ashwag A. Almethen, Maryam A. Alomair

**Affiliations:** a Department of Curriculum and Instruction, College of Education, King Faisal University, Al-ahsa, Saudi Arabia; b King Faisal University, College of Education, Department of Curriculum and Instruction, Al-ahsa, 31982, Saudi Arabia

**Keywords:** Digital citizenship, Teachers' perceptions, Perspectives

## Abstract

This study examined teachers' inclusion of digital citizenship elements in elementary school curricula. Employing a quantitative research design, the study utilized a survey questionnaire as the primary data collection instrument. A quantitative approach was employed in this study because it could efficiently measure the extent and variance of digital citizenship inclusion across diverse teacher demographics, enabling statistically significant comparisons and actionable insights for curriculum development. The sample consisted of 300 elementary school teachers at an educational institution in the Kingdom of Saudi Arabia. The questionnaire assessed the degree to which participants incorporated digital citizenship elements, including respect for self and others, self-education and communication with others, and protecting oneself and others, in curricula. The data were analyzed using descriptive statistics and one-way ANOVA tests. The findings revealed significant differences in teachers’ inclusion of digital citizenship elements based on age, grade level of instruction, and taught subject matter. The study highlights the importance of tailoring digital citizenship education to meet the diverse needs of teachers. These findings contribute to the development of targeted professional development programs and curriculum frameworks that foster responsible and ethical digital participation. The study also holds pedagogical significance by emphasizing the need to account for variations in digital citizenship inclusion across teacher demographics.

## 1 Introduction

Children are exposed to various dangers while browsing social media platforms and the internet. In the digital realm, children may encounter viruses and unsuitable sexual or violent content. They are also vulnerable to financial data theft, information theft, and other security breaches. Children's lack of knowledge surrounding the digital landscape—compounded by their limited understanding of legal, moral, or social repercussions—may also lead them to inadvertently engage in illegal or otherwise unethical activities. In consequence, they may suffer economic, legal, and emotional damages [1]. Research has shown that children mimic the undesirable behaviors of adults while using the internet or electronic applications such as TikTok, Facebook, Twitter, Instagram, and YouTube [2]. This has raised concerns about students' online safety, sparking debate on how to best address these issues [3].

The digital world is rapidly evolving [4]. To protect children from online harm, society must take prudent action [2]. It is imperative that children are trained to become digital citizens, adopting a sense of personal, community-based, and global responsibility. It is equally important that children learn to utilize digital technology for individual empowerment while practicing responsible online behavior [5]. Given the ubiquity of technology both inside and outside of the classroom, digital education has become a necessity; “if we don't take the lead on this issue, they (students) will take the lead” [6] (p. 46). Allowing children to take the lead without proper guidance and education on digital citizenship can lead to potential issues. Depending on the scenario, theu might unknowingly engage in risky online behavior, become targets for exploitation or harassment, and may not know how to handle cyberbullying effectively. Research has shown that a significant percentage of minors are sexually solicited online, primarily by peers and young adults. Ignorance or lack of awareness about these risks could place children in vulnerable situations [6].

Moreover, without proactive teaching and guidance on digital citizenship, schools may find themselves dealing reactively with incidents of online conflicts among students. By addressing digital citizenship proactively, educators can equip students with the necessary skills and knowledge to navigate the digital world responsibly and ethically. Encouraging open dialogue and providing structured instruction on topics like online safety, security, and appropriate online behavior can empower children to make informed decisions and protect themselves from potential dangers [6]. As suggested by Kim and Choi [7], education in digital citizenship should serve as a “new identity card for citizens of the digital era” (p. 158). Such education ensures that users can responsibly enjoy technology, both respecting others’ privacy and safeguarding their own physical, mental, and emotional well-being [4].

Myriad online curricula—endorsing the appropriate, safe, and ethical use of information and technology— have been designed to help students, parents, and educators teach children about digital citizenship. For example, Washington State has provided “digital citizenship curricula” to 76 % of public schools in the United States. These curricula include topics such as online protection, privacy, confidentiality, and cyberbullying. Similarly, the states of California and Texas have advocated for formal education that instructs students on how to address cyberbullying, harmful communications, and psychological and physical threats [8]. In Canada, the Centre for 10.13039/100031019Digital and Media Literacy, MediaSmarts, has developed a set of supporting lesson plans and interactive resources called “Use, Understand & Create: A 10.13039/100031019Digital Literacy Framework for Canadian Schools.” [8].

The framework comprises three eponymous elements: use, understand, and create. “Use” refers to the ability to operate digital devices and software to access and consume digital media content. “Understand” refers to the ability to interpret and analyze digital media content and critically evaluate its quality and credibility. “Create” refers to the ability to produce and share digital media content using various tools and platforms. The UUC framework is designed to be adaptable to diverse age groups and levels of digital literacy. It can also be applied in various contexts such as schools, libraries, homes, and workplaces. The ultimate goal of the framework is to help individuals become confident, competent digital citizens who interact with digital media positively and responsibly [9]. The implementation of such frameworks has been linked with a number of positive outcomes for students. Firstly, students learn to evaluate digital content for reliability and integrity, demonstrating their ability to use critical analysis to discern trustworthy information from misleading sources. Additionally, they acquire digital competence and technological skills through exposure to appropriate digital tools and tasks, becoming proficient in keyboarding and searching efficiently. Moreover, the framework teaches students to respect the intellectual property of digital content, fostering a culture of academic integrity as they understand the importance of citing sources and giving credit to original creators [10]. These positive outcomes can be measured through various assessment methods, including surveys, classroom observations, digital skills assessments, evaluations of students' work quality, and monitoring academic performance and progress. The positive impact of this framework contributes to students' overall growth as critical thinkers, responsible digital citizens, and proficient users of digital tools in the modern digital landscape.

This work explores various definitions of digital citizenship as proposed by the literature. Ribble and Bailey [11] defined digital citizenship as the quality of espousing appropriate and responsible behavior norms when using technology. Ribble's original framework, consisting of nine elements has been summarized into three major categories, including “Respect for Self and Others,” emphasizing digital access, etiquette, and law [12]; “Self-Education and Communication with Others,” including digital communication, literacy, and commerce [3,13]; and “Protecting Oneself and Others,” covering digital rights, responsibilities, security, and health [12,13]. These categories collectively promote responsible and ethical behavior online, urging users to understand legal regulations, practice secure digital habits, and prioritize their mental and physical well-being in the digital realm. Similarly, the International Society for Technology in Education described digital citizenship as using technology safely, legally, and responsibly; promoting a positive attitude towards technology that supports collaboration, learning, and productivity; displaying personal responsibility for lifelong learning; and showing leadership in digital citizenship [14]. In a handbook published by the Council of Europe, digital citizenship is recognized as active and responsible participation in digital communities, competent and positive engagement with digital technologies, involvement in a double process of lifelong learning, and continuous defense of human dignity [15], Emejulu and McGregor [16]. Regarded digital citizenship as a complex process in which individuals and groups committed to social justice engage in discussion and action to build alternative and emancipatory technologies and technological practices. As suggested by Hamayel and Hawamdeh [17], the objective of digital citizenship is, ultimately, to assist younger generations in making appropriate and rational decisions in various digital contexts. From these perspectives, the youth must be educated on core digital citizenship concepts so that they can become responsible online users in the future.

In the context of Saudi Arabia, Alotaibi and Mukred's findings [18] reveal the alarming prevalence of cyber violence, with a significant percentage of students, over 65 percent, reporting victimization and aggression online. This local context underscores the urgency of developing a tailored approach to digital citizenship education that addresses the specific manifestations of online harm faced by students in Saudi Arabia.

Saquib's research [19] has also highlighted the escalating issue of internet addiction among adolescents and young adults in the country. The high prevalence rates indicate the critical need for a comprehensive digital citizenship curriculum that not only focuses on responsible online behavior but also addresses the addictive aspects of internet use as has been the case in other parts of the world. Recognizing and addressing internet addiction within the local context is paramount for promoting a healthy and balanced approach to technology use among Saudi Arabian students.

In Saudi Arabia, other studies have also by Alharbi et al. [20] identified the increased susceptibility of the young demographic in Saudi Arabia to online threats. The cultural dynamics, characterized by a greater distance of power and challenges in open dialogue about online abuse, add complexity to the local context. Understanding these cultural nuances further highlights the need for tailoring digital citizenship education to foster effective communication and trust-building between educators, parents, and students.

In essence, this study responds to the call for context-specific interventions by exploring the perspectives of elementary school teachers in Saudi Arabia. By aligning the global discourse on digital citizenship with the specific challenges outlined in local research, the study aims to contribute to the development of a culturally sensitive and locally relevant digital citizenship framework. This approach recognizes the unique socio-cultural context of Saudi Arabia, ensuring that educators are equipped to address the distinctive digital challenges faced by students in the Kingdom.

Driven by the growing importance of digital citizenship, this study pursues the following objectives. First, it investigates the role of subject specialties in shaping the inclusion of these elements, revealing potential variations across grade levels and disciplines. This is especially important considering that they have a vital role to play in the implementation and shaping digital citizenship education. Lastly, by identifying best practices and areas for improvement, the study aspires to inform the Ministry of Education in modernizing digital citizenship education, ensuring young Saudis are equipped with the necessary skills and knowledge to thrive in the ever-evolving digital landscape.

While prior studies have explored digital citizenship from various perspectives, ranging from adult internet-based community participation to the assessment of digital citizenship elements in college curricula, a crucial gap remains, namely, understanding its integration in elementary school curricula. This study approaches this unexplored territory, using the invaluable lens of teachers' perspectives. The significance of this study is to investigate delves beyond mere inclusion, meticulously examining the depth and nuances of digital citizenship elements within Saudi Arabian elementary programs. This focus on the early years is particularly significant, as its during this formative stage that children develop their foundational understanding of responsible online behavior. Moreover, by exploring the specific challenges faced by educators, often less-accustomed to technology than their students, the study paves the way for targeted interventions and robust training programs that empower teachers to become effective guides on this critical journey. Therefore, this research bridges a vital gap, not only in knowledge but also in empowering a generation of students to navigate the digital world with confidence and responsibility.

Scholars have identified various fundamental components of digital citizenship. Ribble [12], for example, identified nine components, which were also adopted by the ISTE. The iKeepSafe organization—alternatively—presented six elements [14]. These components are outlined in Table 1 below.

**Table 1:** Components of digital citizenship.

Source	Digital Citizenship Components
Ribble [14]	1. Digital access
	2. Digital consumption
	3. Digital communication
	4. Digital literacy
	5. Digital etiquette
	6. Laws and regulations related to digital use
	7. Digital rights and responsibilities
	8. Digital health
	9. Digital security
International Society for Technology in Education	1. Equal digital rights and access for all
	2. Treating others with respect in online environments and avoiding cyberbullying
	3. Not stealing or damaging others' digital work, identity, or property
	4. Appropriate decisions when communicating through digital channels
	5. Using digital tools to advance learning and keeping up with changing technologies
	6. Responsible online purchasing decisions while protecting payment information
	7. Upholding basic digital rights in a digital forum
	8. Protecting personal information from forces that might cause harm
	9. Limiting physical and psychological health risks of technology.

**Table 2:** Socio-demographic characteristics of study participants (n = 300).

		n	%
Gender	Male	144	48.0 %
	Female	156	52.0 %
Experience years	<5	64	21.3 %
	5–10	96	32.0 %
	11–20	85	28.3 %
	21–30	40	13.3 %
	>30	15	5.0 %
Grades levels	Grade 1	39	13.0 %
	Grade 2	59	19.7 %
	Grade 3	51	17.0 %
	Grade 4	101	33.7 %
	Grade 5	82	27.3 %
	Grade 6	116	38.7 %
Subjects titles	Science	47	15.7 %
	Math	33	11.0 %
	Arabic	168	56.0 %
	Islamic studies	69	23.0 %
	Art	42	14.0 %
	Computer	60	20.0 %
	Social studies	35	11.7 %

## 2 Research questions

The main research question of the present study is: To what degree do teachers include various digital citizenship elements in elementary curricula?

The research sub-questions are as follows.

1. Do teachers with varying years of experience have different perceptions of the inclusion of digital citizenship elements in elementary curricula?
2. What are the differences in perceptions of the inclusion of digital citizenship elements in elementary curricula among teachers of different grade levels?
3. Does the perception of digital citizenship elements among teachers vary based on the subjects they teach?

## 3 Hypotheses

The hypotheses of the present study, corresponding with the above research sub-questions, are as follows.

H1 Teachers' inclusion of digital citizenship elements varies significantly based on their years of experience.

H2 Teachers' inclusion of digital citizenship elements varies significantly based on the grade level they teach.

H3 Teachers' inclusion of digital citizenship elements varies significantly based on the subject they teach.

## 4 Literature review

This literature review focuses on unveiling how teachers perceive the inclusion of digital citizenship in the classroom. Understanding these perspectives is crucial given that they have the potential to influence their actions and consequently instructional decisions and the quality of student learning. By delving into existing studies, this review aims to uncover the intricacies of teachers' thoughts and experiences, shedding light on their challenges and beliefs regarding digital citizenship education. This exploration will enrich the understanding of this crucial aspect of education in the Kingdom of Saudi Arabia.

### 4.1 Perspectives on digital citizenship in general

The perspectives of teachers on digital citizenship encompass a range of positive influences and challenges, as illuminated by recent studies. According to Vajen et al. [21] teachers recognize the positive impacts of digital citizenship, particularly in terms of information gathering and increased participation opportunities for students. The advent of digital citizenship facilitates student access to information, enriching their learning experiences. This framework is pertinent in that it illustrates how digital citizenship transforms the learning experiences of students. As such, it is essential for understanding the motivation by teachers for including these elements in their curricula.

Synder [22] further delves into the dual nature of digital citizenship from teachers' perspectives, acknowledging both challenges and benefits associated with digital citizenship. Despite the challenges, such as ensuring effective device utilization, Synder highlights the significant advantages of digital citizenship. These include broadening access to information and resources, establishing a common ground for communication with students, and enhancing the ability of users to respond to events effectively. However, the research underscores the ongoing need for efforts to enhance digital literacy among citizens, emphasizing the importance of understanding how to use technology for analysis, learning, and exploration. This perspective is important for the study because it provides a balanced view. The comprehensive understanding of digital citizenship's positive and negative aspects aligns with the study's aim to understand diverse teacher demographics.

In line with these findings, Pettersson [23] emphasizes that digital citizenship provides a unique opportunity for teachers' professional development. The integration of digital tools and resources offers teachers new avenues for enhancing their skills, staying abreast of educational advancements, and adapting to evolving teaching methodologies. This perspective is also important for the study in that it highlights the professional growth aspect, which is an important point for understanding how digital citizenship education influences or can be tailored to different teacher demographics.

While digital citizenship presents various advantages, Strekalova [24] identifies a number of important challenges, including decreased student motivation, increased screen time, health issues, and unethical activities. Like Synder's study, this is also an important perspective aimed to ensure the literature review addresses critical challenges. It provides a realistic view of the multifaceted nature of digital citizenship, which is cannot only help explain the hesitation on the part of some educators, but also inform tailoring professional development programs.

By synthesizing these perspectives, it becomes evident that teachers recognize the multifaceted nature of digital citizenship. While acknowledging its positive influences on information access and educational engagement, they are also cognizant of the challenges, particularly the ongoing need for digital literacy development. These insights are valuable for understanding how teachers' inclusion of digital citizenship elements varies across demographics.

### 4.2 Teacher perspectives on the value of digital citizenship education for students

Teacher perspectives on the value of digital citizenship education for students are underpinned by recognition of the multifaceted benefits it brings to the evolving landscape of education. As outlined by the American University School of Education [25], teachers play a crucial role in promoting digital citizenship among students for several reasons. The emphasis is placed on enhancing key aspects such as digital literacy, digital etiquette, digital health, and digital security. This comprehensive framework was particularly selected for its holistic approach, which is vital for the development of a well-rounded digital citizenship curriculum tailored to different teacher demographics.

Prasetiyo et al. [26], on the other hand, provides insights from student-teachers who acknowledge the pervasiveness of internet usage in schools and the consequential need for moral guidance in the form of digital citizenship education. They view digital citizenship as a set of principles encompassing knowledge, skills, and appropriate behavior for safe and responsible technology use. Importantly, these student-teachers assert that digital citizenship is a necessary skill for prospective teachers, aligning with the growing demand for character education programs addressing student technology use in schools. This is also an important perspective that gives focus to ethical dimensions, aligning with the need for character education in diverse educational contexts.

Snyder's [22] exploration of the integration of digital citizenship in middle schools reveals a perspective centered on students' future employability. Teachers in this study emphasize the importance of digital citizenship in job preparation, asserting that it contributes to improving students' online reputation and managing their digital footprints. This is an imporant practical aspect that has been included to demonstrate how digital citizenship education enhances students' future employability. As such, it is especially relevant for understanding why some teachers would find it to be a valuable tool not only for themselves but also for preparing students who may need it later on in life.

Drader's insights [27] further underscore the critical importance of digital citizenship in an era where online options are increasingly prevalent, including in education. Educators stress the need for collaboration between schools and families in promoting digital citizenship, emphasizing the benefits of establishing clear boundaries for all stakeholders. This inclusive framework underscores the need for shared responsibility among stakeholders who are in its favor, and consequently aligns with the study's focus on diverse teacher demographics.

In essence, teacher views on the value of digital citizenship education for students converge on the recognition of its multifaceted benefits, ranging from skill development to job preparation and the establishment of clear boundaries in the digital realm. This collective understanding reinforces the integral role of digital citizenship in shaping responsible, informed, and ethical digital citizens for the present and the future.

### 4.3 Challenges and factors affecting the implementation of digital citizenship

In the digital era, the challenge faced by educators is multifaceted, extending beyond the technical aspects of technology use. As highlighted by Martin et al. [28], the increased prevalence of technology necessitates not only the transmission of technical skills but also the cultivation of responsible and ethical behavior online. This challenge is rooted in the dynamic nature of the digital landscape, where students must navigate a vast array of online platforms. Educators grapple with the task of guiding students toward not just proficient technology use but also fostering a sense of responsibility and appropriateness in their digital interactions. This perspective is valuable for its emphasis on ethical considerations, which are critical for comprehensive digital citizenship education tailored to different demographic factors. It can also play a vital role to help understand why different groups of educators perceive digital citizenship as they do.

Snyder's exploration [22] on the other hand reveals a critical challenge related to access barriers and filtering mechanisms in the digital realm. While digital citizenship education aims to empower students with digital skills and knowledge, the presence of access barriers, such as limited availability of technology and internet connectivity, can hinder the realization of these goals. Moreover, the use of website blocking and content filtering in schools can inadvertently restrict students' exposure to diverse perspectives and global digital citizenship initiatives. The study directly addresses practical barriers to consider, providing a realistic view of implementation challenges across different educational contexts.

Vang's study [29] sheds light on a crucial aspect of the digital citizenship landscape, highlighting the role of educators and families. While the focus is often on students as digital citizens, Vang highlights the indispensable role of educators in modeling digital citizenship behaviors. However, this task is hindered by a lack of access and resources for educators. The absence of adequate support mechanisms for educators can impede their own growth as digital citizens, limiting their ability to effectively guide and mentor students in responsible online behavior. This underscores the need for adequate support structures, which is crucial for successful implementation across different teacher demographics. It also indicates that some of the perspectives held go beyond what educators can control.

Liu and Liu's research [30] delves into the impact of demographic factors on the effective implementation of digital citizenship. The study reveals that various demographic factors, such as the rural-urban divide and age-related differences among teachers, can significantly influence the adoption and integration of digital citizenship principles. Rural teachers, influenced by their preference for offline interactions, face challenges in embracing digital devices and participating in online communities. Additionally, the study highlights that older teachers, with less experience in digital citizenship, may struggle to impart this knowledge to students. The study also identifies funding constraints as a substantial hindrance to the effective implementation of digital citizenship, particularly in underdeveloped areas. The provision of quality digital citizenship education requires financial resources for training, updating technological infrastructure, and ensuring access to relevant digital tools. The lack of adequate funding presents a barrier to creating an environment conducive to the successful integration of digital citizenship initiatives. Overcoming these financial constraints becomes imperative for establishing an inclusive and comprehensive digital citizenship education framework that can thrive across diverse economic contexts. The perspective highlights the diversity of challenges that are faced by different demographic groups and thus advocates for tailored strategies in digital citizenship education. This aligns with the study's focus on age, grade level, and subject area.

Overall, the challenges in implementing digital citizenship effectively are complex and multifaceted, involving not only conceptual and educational aspects but also practical, infrastructural, and socio-economic considerations. Acknowledging and addressing these challenges are essential steps toward fostering a robust digital citizenship education framework that equips both educators and students for responsible and ethical engagement in the digital age.

### 4.4 Teachers’ perceptions and perspectives on effective implementation

The effective implementation of digital citizenship in educational settings is a nuanced process, influenced by teachers' perceptions and experiences. Insights from various studies shed light on key considerations that impact the successful integration of digital citizenship education.

Emejulu and McGregor's study [16] provides a comprehensive view of high school teachers' perceptions regarding the importance of the nine tenets of digital citizenship. While there was a unanimous agreement on the significance of all these principles, differences emerged, particularly in digital communication and digital etiquette between male and female educators. This underscores the necessity for comprehensive education for all teachers to ensure a unified approach in teaching these critical aspects of digital citizenship. Notably, the study identified a scarcity of formal digital citizenship education in schools. Emejulu and McGregor [16] suggests that establishing a formal curriculum can increase the frequency of instruction, fostering a culture of continued implementation. This aligns with Vang's findings [29], emphasizing the need for educators' support in digital citizenship classes, including sufficient time allocation and access to resources and funds to enhance frequent instruction.

Synder's study [22] introduces a unique perspective on effective implementation by highlighting the role of global collaborative projects and social media. Teachers noted that these approaches helped overcome barriers to digital citizenship, fostering positive attitudes and behaviors among students. Global collaborative projects not only provided students with exposure to diverse cultures but also encouraged them to consider the welfare of others online. This positive influence contributed to the modification of online behavior, aligning with the principles of digital citizenship. The study supports the inclusion of global collaborative projects in curricula as a means to enhance digital citizenship education. The innovative approach also effectively showcases alternative methods of enhancing digital citizenship education, relevant for diverse educational contexts.

Haase's study [31] delves into elementary teachers' perspectives on the value of digital citizenship. The majority of teachers agreed on the importance of elements such as digital etiquette, digital security and privacy, and digital health and welfare. Importantly, the study identified that teachers with more experience in virtual environments reported higher levels of implementation and had more positive attitudes towards individual digital citizenship elements. This emphasizes the correlation between experience and effective implementation, suggesting the need for ongoing professional development and support to enhance teachers' appreciation and successful integration of digital citizenship education. This perspective serves to not only highlighting the need for ongoing professional development, but also demonstrating how and why it is crucial for effective implementation across different teacher demographics.

Based on these findings, teachers' perceptions of effective implementation of digital citizenship underscore the importance of comprehensive education, formal curriculum development, support structures, and the integration of innovative approaches such as global collaborative projects. Moreover, the positive correlation between experience and successful implementation emphasizes the need for continued professional development to foster a culture of digital citizenship within educational environments.

### 4.5 The perceptions and perspectives of Saudi Arabia teachers on digital citizenship and digital citizenship education

Understanding the perceptions and perspectives of Saudi Arabian teachers on digital citizenship and digital citizenship education is essential for tailoring educational strategies to the cultural and contextual nuances of the region. Insights from Alqirnas [32] and Alqahtani [33] provide valuable perspectives on these matters.

Alqirnas [32] delves into the perspectives of primary teachers in Saudi Arabia, revealing positive implementations of the concept of digital citizenship and its various dimensions. Notably, the study found that age had no significant impact on digital citizenship dimensions among primary teachers. However, an intriguing correlation emerged—primary teachers with over 10 years of technological experience demonstrated a higher level of knowledge regarding digital citizenship, particularly in areas such as digital communication and digital etiquette. This aligns with global findings that suggest age and experience are crucial factors in digital citizenship instruction and implementation. Al-Zahrani's [34] identification of factors influencing digital citizenship, such as computer experience, daily technology use, students' attitudes toward the Internet, and computer self-efficacy, further supports the importance of experience in shaping attitudes and practices in digital citizenship education. Educators with more experience are likely to instill similar experiences and attitudes in their students. This perspective is included to illustrate the significance of experience, aligning with global trends and the study's focus on teacher demographics. Experience is especially an important factor that can help explain why some educators are more likely to be for the implementation while others, based on their experience, may be cautious.

Alqahtani's study [33] among male participants in Saudi Arabia also indicates positive attitudes and awareness of digital citizenship. The study demonstrates an understanding of key factors associated with digital citizenship, such as respect, education, and protection, aligning with the categorization by Ribble. Despite the positive attitudes, the study highlights a gender limitation as only male perspectives were considered, leaving a gap in understanding the female perspective on digital citizenship. Moreover, participants expressed the importance of aligning electronic teaching aids with Islamic culture and religion to reduce exposure to harmful content. This cultural consideration emphasizes the need for culturally sensitive approaches in digital citizenship education, relevant for understanding how different demographics in Saudi Arabia perceive and respond to digital citizenship.

The perspectives of Saudi Arabian teachers on digital citizenship reveal positive attitudes and awareness, particularly among those with more technological experience. The correlation between experience and knowledge in digital citizenship aligns with global trends. However, cultural considerations, such as aligning teaching aids with Islamic values, underscore the impact that culture has on how concepts like digital citizenship is perceived, implemented, and utilized.

## 5 Methods

### 5.1 Study design

This study employed a quantitative research design to investigate teachers' perceptions of digital citizenship in elementary curricula. The research utilized a survey questionnaire as the primary data collection instrument. The choice of a quantitative research design for this study was driven by its suitability for objective, quantifiable data analysis, particularly when dealing with a large sample size like 300 teachers. This approach allows for effective measurement and statistical analysis of the extent to which digital citizenship elements are incorporated into curricula, aligning with the study's aim to identify specific trends and patterns across various teacher demographics. Quantitative methods provide the robustness required for hypothesis testing and generalizability of findings, essential for this study's objectives. While mixed methods offer richer, more nuanced data, a quantitative approach was deemed more efficient and appropriate for the scope and aims of this particular research. This study was approved by the Ethics Committee of King Faisal University, with ethics approval reference [KFU-REC-2023-MAR-ETHICS715].

### 5.2 Participants

The participants comprised teachers from various educational institutions, totaling 300 individuals. This sample included both female and male teachers, with a representation of diverse experience levels. The range of experience spanned from less than 5 years to over 30 years, with a noticeable number of participants having 5–10 years and 11–20 years of teaching experience. The sample also covered teachers from different grade levels, from Grade 1 through Grade 6. Additionally, these teachers were from various subject specializations, with many teaching Arabic and Islamic studies, among others.

### 5.3 Materials

The questionnaire was divided into two main sections: demographic information and digital citizenship elements. The demographic section gathered basic information about the participants, including their gender, years of teaching experience, grade levels taught, and the subjects they specialized in. The second section, focusing on digital citizenship, was based on Ribble's “Nine Elements of Digital Citizenship.” This part of the questionnaire comprised 45 items, assessed using a 5-point Likert scale ranging from 1 (Strongly Disagree) to 5 (Strongly Agree). These items were evenly distributed across three key categories: Respect for Self and Others (15 items), Self-Education and Communication with Others (15 items), and Protecting Oneself and Others (15 items). The questionnaire was refined for clarity and relevance through a pilot study with 30 teachers and expert feedback. Its reliability was confirmed with a high Cronbach's alpha score of 0.913, ensuring its effectiveness in measuring the intended constructs of digital citizenship within educational settings.

### 5.4 Pilot study

Before administering the final version of the questionnaire, a pilot study was conducted among thirty teachers in educational institutions. Modifications were made to the questionnaire based on the feedback from a committee of twelve educational experts, ensuring the questionnaire was easily understandable and clear for the target population group. A reliability analysis was performed using Cronbach's alpha to assess the internal consistency of the study variables. The Cronbach's alpha was 0.913. High level of internal consistency was found for the questionnaire, indicating that the questions within the questionnaire are highly related and measure the same construct.

The survey questionnaire utilized in the study, based on Ribble's “Nine Elements of Digital Citizenship,” encompassed a total of 45 items. These items were distributed across the nine elements to assess various aspects of digital citizenship. Here is the breakdown of the number of questions assigned to each of Ribble's nine elements: The Nine Elements of Digital Citizenship are as follows.

1. Digital Access: This element focuses on ensuring equitable access to technology and digital resources. It involves bridging the digital divide and promoting equal opportunities for all individuals to benefit from digital tools and information.
2. Digital Commerce: Digital commerce pertains to responsible and ethical participation in online economic activities. It involves understanding online transactions, e-commerce practices, and consumer rights, as well as being aware of online scams and deceptive practices.
3. Digital Communication: This element emphasizes the importance of effective and respectful communication in digital environments. It encompasses concepts such as appropriate online behavior, responsible use of digital communication tools (email, messaging, social media), and the potential consequences of online interactions.
4. Digital Literacy: Digital literacy refers to the ability to find, evaluate, and use digital information effectively. It involves developing critical thinking skills, media literacy, and the ability to assess the credibility and reliability of online sources.
5. Digital Etiquette: Digital etiquette encompasses the social norms and guidelines for appropriate behavior in online interactions. It involves understanding and practicing online etiquette, respecting others' privacy, and promoting a positive digital culture.
6. Digital Law: This element focuses on understanding and adhering to the legal frameworks and regulations governing digital spaces. It includes topics such as copyright, intellectual property rights, data protection, and cybercrime prevention.
7. Digital Rights and Responsibilities: The element of digital rights and responsibilities highlights the rights and freedoms individuals have in the digital realm, as well as the corresponding responsibilities. It involves promoting digital rights such as freedom of speech, privacy, and access to information, while also recognizing the responsibility to respect others' rights and contribute positively to the online community.
8. Digital Health and Wellness: This element addresses the well-being and health considerations related to digital technology use. It encompasses aspects such as maintaining a healthy balance between online and offline activities, managing screen time, addressing digital addiction, and fostering positive digital well-being.
9. Digital Security: Digital security focuses on the protection of digital systems, data, and privacy. It involves understanding cybersecurity measures, safe online practices, password security, and safeguarding personal information from online threats.

Ribble's Elements Tool provides a comprehensive framework for educators, parents, and policymakers to guide the development of digital citizenship education programs and initiatives (Ribble, 2011). By integrating these elements into curricula and fostering their understanding among students, individuals can navigate the digital world responsibly, ethically, and safely. The tool serves as a valuable resource in promoting digital citizenship skills and empowering individuals to become responsible digital citizens in today's interconnected world.

### 5.5 Data analysis

The collected data was revised, coded in MS Excel, and analyzed by IBM SPSS 26 software. Descriptive analysis based on the frequency and percent distribution was conducted for all variables. A one-way ANOVA test was conducted according to the categories of the data. This study adopted a significance level of less than 5 % (p < 00.05). The ranks in Table 3, Table 4, Table 5 represent the mean scores of respondents' agreement on the respective items, ordered from highest to lowest mean.

**Table 3:** *Results ofRespect forSelf andOthers (R)*

	SD^a^	D	N	A	SA	Mean	Std. Dev.	Degree	Rank
R1	2(0.67)	100(33.33)	51(17)	94(31.33)	53(17.67)	3.32	1.13	N	12
R2	2(0.67)	90(30)	38(12.67)	98(32.67)	72(24)	3.49	1.17	A	6
R3	2(0.67)	91(30.33)	41(13.67)	99(33)	67(22.33)	3.46	1.16	A	7
R4	4(1.33)	95(31.67)	31(10.33)	108(36)	62(20.67)	3.43	1.17	A	8
R5	2(0.67)	96(32)	40(13.33)	98(32.67)	64(21.33)	3.42	1.16	A	9
R6	2(0.67)	48(16)	44(14.67)	126(42)	80(26.67)	3.78	1.03	A	3
R7	0(0)	50(16.67)	40(13.33)	138(46)	72(24)	3.77	0.99	A	4
R8	4(1.33)	41(13.67)	54(18)	128(42.67)	73(24.33)	3.75	1.02	A	5
R9	0(0)	44(14.67)	46(15.33)	138(46)	72(24)	3.79	0.97	A	1
R10	4(1.33)	43(14.33)	48(16)	126(42)	79(26.33)	3.78	1.04	A	2
R11	2(0.67)	95(31.67)	54(18)	104(34.67)	45(15)	3.32	1.09	N	13
R12	2(0.67)	101(33.67)	41(13.67)	96(32)	60(20)	3.37	1.162	N	11
R13	6(2)	105(35)	46(15.33)	94(31.33)	49(16.33)	3.25	1.157	N	15
R14	2(0.67)	96(32)	46(15.33)	90(30)	66(22)	3.41	1.169	A	10
R15	6(2)	95(31.67)	54(18)	90(30)	55(18.33)	3.31	1.157	N	14
Overall mean						3.51	A		

a SD=Strongly Disagree, D = Disagree, N=Neutral, A = Agree, SA= Strongly Agree.

**Table 4:** Results of *Self-Education/Connecting withOthers (S)*

	SD	D	N	A	SA	Mean	Std. Dev.	Degree	Rank
S1	0(0)	37(12.33)	51(17)	131(43.67)	81(27)	3.85	0.96	A	6
S2	0(0)	36(12)	45(15)	131(43.67)	88(29.33)	3.90	0.96	A	2
S3	0(0)	38(12.67)	56(18.67)	121(40.33)	85(28.33)	3.84	0.98	A	7
S4	4(1.33)	63(21)	43(14.33)	125(41.67)	65(21.67)	3.61	1.08	A	9
S5	6(2)	61(20.33)	49(16.33)	119(39.67)	65(21.67)	3.59	1.10	A	10
S6	2(0.67)	48(16)	37(12.33)	133(44.33)	80(26.67)	3.80	1.03	A	8
S7	4(1.33)	42(14)	25(8.33)	136(45.33)	93(31)	3.91	1.03	A	1
S8	2(0.67)	40(13.33)	39(13)	129(43)	90(30)	3.88	1.01	A	3
S9	2(0.67)	42(14)	32(10.67)	145(48.33)	79(26.33)	3.86	0.99	A	5
S10	4(1.33)	38(12.67)	39(13)	131(43.67)	88(29.33)	3.87	1.02	A	4
S11	6(2)	108(36)	34(11.33)	89(29.67)	63(21)	3.32	1.22	N	11
S12	6(2)	115(38.33)	39(13)	84(28)	56(18.67)	3.23	1.20	N	14
S13	8(2.67)	112(37.33)	50(16.67)	75(25)	55(18.33)	3.19	1.2	N	15
S14	7(2.33)	110(36.67)	27(9)	97(32.33)	59(19.67)	3.30	1.22	N	12
S15	8(2.67)	101(33.67)	39(13)	99(33)	53(17.67)	3.29	1.18	N	13
overall mean						3.63	A

**Table 5:** Results of *ProtectingOneself andOthers (P)*

	SD	D	N	A	SA	mean	Std. Dev.	Degree	Rank
P1	2(0.67)	102(34)	29(9.67)	107(35.67)	60(20)	3.40	1.17	A	7
P2	4(1.33)	106(35.33)	31(10.33)	104(34.67)	55(18.33)	3.33	1.18	N	12
P3	5(1.67)	100(33.33)	33(11)	99(33)	63(21)	3.38	1.2	N	10
P4	5(1.67)	95(31.67)	38(12.67)	100(33.33)	62(20.67)	3.40	1.18	A	8
P5	6(2)	96(32)	37(12.33)	86(28.67)	75(25)	3.43	1.23	A	6
P6	7(2.33)	99(33)	34(11.33)	91(30.33)	69(23)	3.39	1.23	N	9
P7	8(2.67)	101(33.67)	41(13.67)	88(29.33)	62(20.67)	3.32	1.21	N	13
P8	7(2.33)	109(36.33)	55(18.33)	76(25.33)	53(17.67)	3.20	1.18	N	15
P9	6(2)	92(30.67)	46(15.33)	104(34.67)	52(17.33)	3.35	1.15	N	11
P10	9(3)	101(33.67)	46(15.33)	98(32.67)	46(15.33)	3.24	1.16	N	14
P11	1(0.33)	91(30.33)	43(14.33)	99(33)	66(22)	3.46	1.15	A	4
P12	4(1.33)	82(27.33)	46(15.33)	100(33.33)	68(22.67)	3.49	1.16	A	2
P13	3(1)	82(27.33)	46(15.33)	99(33)	70(23.33)	3.50	1.15	A	1
P14	4(1.33)	89(29.67)	41(13.67)	97(32.33)	69(23)	3.46	1.18	A	5
P15	9(3)	76(25.33)	48(16)	101(33.67)	66(22)	3.46	1.18	A	3
overall mean						3.39	N		

## 6 Results

### 6.1 Demographic variables

Table 2 reveals a relatively balanced representation for the gender with 52 % of teachers being female and 48 % male. In terms of experience years, the majority of teachers fall into the 5–10 years of experience category, comprising 32 % of the total. Additionally, 28.3 % of teachers have 11–20 years of experience. Examining the distribution of teachers across different grade levels, Grade 6 has the highest number of teachers, with 38.7 % specializing in this grade. Similarly, Grade 4 has a significant representation with 33.7 % of teachers. Conversely, Grades 1 and 3 have lower proportions of teachers. Regarding the subjects taught by teachers, the table offers insights into their areas of specialization. Arabic stands out as the most commonly taught subject, with 56 % of teachers specializing in it. Islamic studies follows closely with 23 % of teachers focusing on this subject, indicating its importance as a core area of instruction. Science, Art, and Computer are taught by 15.7 %, 14 %, and 20 % of teachers, respectively, highlighting the value placed on these subjects in the curriculum. On the other hand, Math and Social Studies have the lowest representation, with 11 % and 11.7 % of teachers, respectively.

### 6.2 Respect for self and others (R)

Respect for self and others (R) contains 15 question on the questionnaire (from R1-R15) (see appendix A). Table 3 shows that the overall mean for this factor was 3.51 indicating agreement on the factor of respect for self and other. The top three items with the highest mean scores indicated a strong consensus on the importance of respect, the first item was item (R9) achieved the highest mean score of 3.79, followed by item (R10) received a mean score of 3.78 and ranked second, the third item was (R6) also received a mean score of 3.78, securing the third rank. This result reinforces the general consensus on the significance of respect for self and other.

Conversely, the three items with the lowest mean scores demonstrated comparatively lower levels of agreement were item (R13) which had the lowest mean score of 3.25 among all items, indicating a weaker consensus regarding respect. It ranked fifteenth in the table, followed by item (R15) which received a mean score of 3.31 and ranked fourteenth, followed by item (R11) which obtained a mean score of 3.32, placing it thirteenth in the rankings. Table 3 represents these results.

### 6.3 Self-education/connecting with others (S)

Self-education/connecting with others (S) contains 15 question on the questionnaire (from S1–S15) (see appendix A). Table 4 shows that the overall mean for this factor was 3.63 indicating agreement on the factor of (Self education/communication with others). The top three items with the highest mean scores indicated a strong consensus on the importance of respect, the first item was item (S7) achieved the highest mean score of 3.91, followed by item (S2) received a mean score of 3.90 and ranked second, the third item was (S8) also received a mean score of 3.88, securing the third rank. This result reinforces the general consensus on the significance of Self education/communication with others.

Conversely, the three items with the lowest mean scores demonstrated comparatively lower levels of agreement were item (S13) which had the lowest mean score of 3.19 among all items, indicating a weaker consensus regarding respect. It ranked fifteenth in the table, followed by item (S12) which received a mean score of 3.23 and ranked fourteenth, followed by item (S15) which obtained a mean score of 3.29, placing it thirteenth in the rankings. Table 4 represents these results.

### 6.4 Protecting oneself and others (P)

Protecting oneself and others (P) contains 15 question on the questionnaire (from P1–P15) (see appendix A). Table 5 shows that the overall mean for this factor was 3.39 indicating a neutral agreement on the factor of (Protecting oneself and others). The top three items with the highest mean scores indicated a strong consensus on the importance of respect, the first item was item (P13) achieved the highest mean score of 3.50, followed by item (P12) received a mean score of 3.49 and ranked second, the third item was (P15) also received a mean score of 3.46, securing the third rank.

Conversely, the three items with the lowest mean scores demonstrated comparatively lower levels of agreement were item (P8) which had the lowest mean score of 3.20 among all items, indicating a weaker consensus regarding respect. It ranked fifteenth in the table, followed by item (P10) which received a mean score of 3.24 and ranked fourteenth, followed by item (P7) which obtained a mean score of 3.32, placing it thirteenth in the rankings. Table 5 represents these results.

### 6.5 Demographic variables and respect for self and others

Regarding experience years, Table 6 shows that age group (21–30) years was significantly highest in mean for Digital Etiquette, and Digital Law (p = 0.0001, and 0.002 respectively).

**Table 6:** Demographic variables and respect for self and others

			N	Mean	SD	p-value
Experience years	Digital Etiquette	<5	64	17.4	5.2	0.000
		5–10	96	15.4	5.5	
		11–20	85	17.1	5.6	
		21–30	40	20.4	3.0	
		>30	15	18.0	4.9	
	Digital Law	<5	64	16.9	5.1	0.002
		5–10	96	15.2	5.6	
		11–20	85	16.6	5.5	
		21–30	40	19.3	4.3	
		>30	15	18.2	5.0	
grade 3	Digital Etiquette		51	13.31	3.99	0.0001
grade 4	Digital Etiquette		101	18.37	5.45	0.0001
grade 5	Digital Access		82	20.33	3.94	0.0001
grade 6	Digital Law		116	18.10	4.97	0.0001
Arabic	Digital Law		168	19.05	4.58	0.0001
Islamic studies	Digital Access		69	17.45	4.56	0.003
Computer	Digital Law		60	15.25	5.15	0.043

Regarding grades, teachers who were teaching to grade 3 and 4 were significantly difference than other teachers who teach to other grades in Digital Etiquette (p = 0.0001), and teacher who were teaching to grade 5 were significantly difference than others in Digital Access (p = 0.0001).

Furthermore, regarding teaching subjects, teachers who were teaching Arabic subject were significantly difference in Digital Law than others (p = 0.0001), teachers who were teaching Islamic studies subject were significantly difference in Digital Access than others (p = 0.003), also teachers who were teaching computer subject were significantly difference in Digital Law than others (p = 0.043).

### 6.6 Demographic variables and self-education/connecting with others

Regarding experience years, Table 7 shows that age group (21–30) years was significantly highest in mean for Digital Literacy, and Digital Commerce (p = 0.031, and 0.001 respectively).

**Table 7:** Demographic variables and self-education/connecting with others

			N	Mean	SD	p-value
Experience years	Digital Literacy	<5	64	18.5	4.2	0.031
		5–10	96	19.2	5.0	
		11–20	85	19.5	4.6	
		21–30	40	21.1	3.1	
		>30	15	17.4	6.8	
	Digital Commerce	<5	64	16.3	4.8	0.001
		5–10	96	14.9	5.8	
		11–20	85	16.4	6.0	
		21–30	40	19.5	5.2	
		>30	15	16.6	6.6	
grade 3	Digital Communication		51	16.43	4.41	0.0001
grade 4	Digital Communication		101	20.15	4.22	0.0001
grade 5	Digital Commerce		82	19.32	5.11	0.0001
grade 6	Digital Commerce		116	17.72	5.32	0.0001
Arabic	Digital Literacy		168	20.60	3.91	0.0001

Regarding grades, teachers who were teaching to grade 3 and 4 were significantly difference than other teachers who teach to other grades in Digital Communication (p = 0.0001), and teacher who were teaching to grade 5 and 6 were significantly difference than others in Digital Commerce (p = 0.0001).

Furthermore, regarding teaching subjects, teachers who were teaching Arabic subject were significantly difference in Digital Literacy than others (p = 0.0001).

### 6.7 Demographic variables and protecting oneself and others

Regarding experience years, Table 8 shows that age group (21–30) years was significantly highest in mean for Digital Rights, Digital Security, and Digital Health and Wellness (p = 0.0001).

**Table 8:** Demographic variables and protecting oneself and others

			N	Mean	SD	p-value
Experience years	Digital Rights	<5	64	17.6	5.2	0.000
		5–10	96	15.1	5.9	
		11–20	85	16.8	6.0	
		21–30	40	20.0	3.6	
		>30	15	18.7	5.1	
	Digital Security	<5	64	16.5	5.0	0.000
		5–10	96	15.0	5.7	
		11–20	85	16.3	5.8	
		21–30	40	19.4	4.4	
		>30	15	18.9	5.1	
	Digital Health and Wellness	<5	64	17.2	5.1	0.000
		5–10	96	15.8	5.8	
		11–20	85	17.4	5.6	
		21–30	40	20.6	3.4	
		>30	15	18.9	5.1	
grade 3	Digital Rights		51	13.47	5.00	0.0001
grade 4	Digital Health and Wellness		101	18.90	5.54	0.0001
grade 5	Digital Rights		82	20.04	4.98	0.0001
grade 6	Digital Rights		116	18.41	5.30	0.0001
	Digital Security		116	17.69	5.36	0.0001
Arabic	Digital Health and Wellness		168	19.72	4.51	0.0001

Regarding grades, teachers who were teaching to grade 3,5, and 6 were significantly difference than other teachers who teach to other grades in Digital Rights (p = 0.0001), and teacher who were teaching to grade 4 were significantly difference than others in Digital Health and Wellness (p = 0.0001).

Furthermore, regarding teaching subjects, teachers who were teaching Arabic subject were significantly difference in Digital Health and Wellness than others (p = 0.0001).

## 7 Discussion

This study aimed to examine the extent to which elements of digital citizenship are incorporated into elementary curricula in Saudi Arabia, based on teachers' self-reported perspectives. The research questions focused on how teachers’ years of experience, taught grade level, and subject area influence their inclusion of key components of digital citizenship education.

### 7.1 Teachers’ experience and digital citizenship

The absence of a significant correlation between teaching experience and the incorporation of digital citizenship elements in this study presents an interesting contrast to existing literature. Previous research often posits that novice teachers, due to their relatively recent exposure to technology in their formative years, might be more adept at integrating digital tools and concepts into their teaching [[35], [36], [37]]. In contrast, veteran teachers, who may have started their careers before the digital revolution in education, often face more significant challenges in adapting to technology-rich teaching environments [38,39]. However, the findings from the Saudi Arabian context suggest a different narrative, where experience does not necessarily equate to proficiency or confidence in embedding digital citizenship in education.

One explanation for this could be the universal challenge posed by the ever-evolving nature of digital technologies and online environments. The rapid pace of change in the digital world means that what was learned a few years ago may no longer be applicable or sufficient today. This continuous evolution necessitates an ongoing learning process for all teachers, regardless of their years of experience [[40], [41], [42]]. As such, professional development in digital citizenship should be viewed as a continuous journey rather than a one-time training event. This approach would ensure that all educators, regardless of their experience level, remain updated with the latest digital trends and educational technologies [43].

Moreover, the findings highlight the potential benefits of fostering collaborative communities of practice among educators. Such communities could provide platforms for experienced teachers to share their pedagogical expertise and classroom management skills, while novice teachers could contribute their up-to-date knowledge of the latest digital tools and trends [44]. This reciprocal exchange of knowledge and experience can lead to more effective and innovative digital citizenship education strategies. The collaborative sharing of expertise not only enriches the teaching practice but also creates a supportive network that can help educators navigate the complexities of teaching digital citizenship [45].

Additionally, the study's findings suggest that in-service training and professional development programs in Saudi Arabia should be designed to cater to the diverse needs of teachers at different stages of their careers [46]. Tailoring these programs to address specific gaps or needs can enhance their effectiveness. For instance, while novice teachers might benefit more from workshops focusing on integrating digital citizenship into curriculum planning and classroom management, veteran teachers might require more support in using specific digital tools and platforms [47].

### 7.2 Grade level and digital citizenship

The study's findings that teachers of grades 3 and 4 place greater emphasis on digital communication skills are particularly significant. This emphasis is well-aligned with developmental milestones for children aged 8–10, as identified by developmental psychologists like Santrock. At this age, children are developing critical communication and collaboration skills, making this an ideal time to introduce and reinforce concepts like online etiquette and ethical digital interaction. These foundational skills are crucial for students to engage positively and responsibly in digital spaces.

The integration of digital citizenship at this stage has the potential for profound long-term impacts on students' digital lives. By focusing on communication skills, teachers are preparing students not only for immediate safe online interactions but also for future challenges they may face in increasingly digital societies [48]. Lessons that emphasize respectful online communication, understanding digital footprints, and the consequences of digital actions play a significant role in shaping responsible digital citizens [49].

The heightened focus on digital commerce concepts in grades 5 and 6 is another key finding of the study. This focus dovetails with curricular objectives that are increasingly incorporating elements of financial literacy and basic economic principles at these grade levels. The inclusion of topics like online transactions, consumer rights, and data privacy in the curriculum is a timely response to the growing digital economy and its impact on students' lives [50].

As students in these grades are beginning to experience greater independence and more engagement in online activities, it becomes crucial to equip them with the skills and knowledge to navigate these digital environments safely and responsibly. This includes understanding the nuances of online shopping, recognizing and protecting against potential online frauds and scams, and being aware of their rights as digital consumers. Teaching these concepts helps bridge the gap between classroom learning and real-world application, preparing students for the complexities of the digital world they will continue to encounter and engage with as they grow [51].

### 7.3 Subject area and digital citizenship

In the context of Arabic language education, the study revealed a strong emphasis on digital literacy and digital health/wellness. This finding aligns with the intrinsic nature of language instruction, which not only involves the development of linguistic skills but also critical thinking and information analysis. Arabic teachers, therefore, play a pivotal role in shaping how students interact with and interpret online content [52]. Their focus extends beyond mere language proficiency to encompass a broader educational goal – equipping students with the skills to navigate the digital world responsibly. This includes teaching students how to evaluate online sources critically, understanding the ethical implications of digital content, and promoting balanced technology use to safeguard physical and mental health. Such an approach is crucial in an era where information is abundant and not always reliable, underscoring the role of language teachers in fostering digital literacy [53].

Teachers of Islamic studies prioritizing digital access is a significant finding, reflecting the integration of faith-based values into digital citizenship education. In Islamic studies, the emphasis on equity and inclusion can be seamlessly extended to the digital realm [54]. These teachers are uniquely positioned to impart the importance of ensuring equal digital opportunities for all, regardless of socio-economic backgrounds. This approach resonates with the ethical principles of fairness and inclusivity inherent in Islamic teachings. By emphasizing digital access, Islamic studies teachers contribute to a more equitable digital environment where all students have the opportunity to engage and learn, thus promoting a sense of community and shared responsibility in the digital world [55].

The enhanced focus of computer science teachers on legal and ethical issues, including digital security, intellectual property, and digital rights, is a logical extension of their subject matter. Given the technical nature of computer science, these educators are well-equipped to delve into the intricacies of online safety, data protection, and the legal ramifications of digital actions [56]. Their role is crucial in preparing students to understand and navigate the complex legal landscape of the digital world. This includes educating students on issues such as cybersecurity, protecting intellectual property, and understanding user rights and responsibilities online. By doing so, computer science teachers are not just imparting technical knowledge but also instilling a sense of digital responsibility and ethics among students [57].

The study's findings underscore the need for a collaborative, cross-disciplinary approach to digital citizenship education. Each subject area offers unique opportunities to integrate digital citizenship concepts, and a cohesive strategy can ensure that students receive a comprehensive education in this area. For example, science and math classes can focus on data literacy and analysis, equipping students with skills to interpret and utilize digital data effectively. Social studies classes can incorporate discussions on the societal impacts of technology, exploring topics like digital democracy, online activism, and the ethical implications of technological advancements [58]. By integrating digital citizenship across various subjects, educators can provide students with a well-rounded understanding of how to navigate the digital world responsibly and ethically, preparing them for the challenges and opportunities of the 21st century [59].

## 8 Implications

The results have important implications for policy makers, educators, curriculum developers, and cultural considerations. As online engagement grows, education must adapt to equip students with the skills and ethical foundations to safely navigate the digital world [60,61]. professional development programs should differentiate training based on teachers’ backgrounds and experience levels. Younger teachers may be more receptive to emerging technologies and digital platforms, while older teachers possess deeper pedagogical expertise to integrate digital citizenship across subjects. Recognizing these complementary strengths can enrich training outcomes [62,63].

Additionally, digital citizenship instruction should be tailored for specific grade levels based on the developmental needs of students. Early childhood and lower elementary grades may focus more on digital etiquette and online safety. Upper elementary and middle school can delve into topics like privacy, cyberbullying, and media literacy as students increase technology use. High school would prepare students for civic participation, cybersecurity, and legal/ethical issues they may encounter in higher education or the workplace [64,65].

Furthermore, digital citizenship education cannot fall solely under the purview of technology/computer teachers. A cross-curricular approach that embeds digital citizenship across diverse subjects is essential [64]. This requires coordination between administrators, teachers, and technology specialists when designing curricula, lesson plans, and activities. Digital citizenship should also be explicitly included in curriculum frameworks and standards at the national level to clearly signal its importance [66,67].

The local Saudi context additionally necessitates balancing global digital citizenship norms with cultural values. As Alqirnas [32] noted, teachers must navigated this when addressing topics like free expression, gender dynamics, and privacy. Constructive dialogue between education leaders, community members, and teachers can produce guidance on sensitively integrating digital citizenship in a culturally-responsive manner.

## 9 Limitations

While this study contributes to understanding how teachers perceive the inclusion of digital citizenship elements in elementary curricula, it is important to acknowledge certain limitations. The study was conducted within a specific educational institution, which limits the generalizability of the findings to a broader population. As such, the findings may not be representative of all elementary curricula or teacher populations. The data collected was developed from self-reported responses from teachers, which may be subject to social desirability bias or inaccuracies. For instance, the veracity of participants' responses might have been compromised by a desire to modify their responses to appear socially acceptable. In addition, the accuracy of teachers’ responses may have been affected by human fallacy in recall or perception.

Furthermore, the study focused primarily on teachers’ experiences, taught grade level, and subject of instruction as variables of interest. However, other pertinent factors such as professional development opportunities, technological resources, and institutional policies were not examined; such factors may additionally influence teachers' inclusion of digital citizenship elements. Furthermore, the study did not explicitly consider cultural or contextual factors that may influence teachers' perceptions of digital citizenship; different cultural norms and educational systems may shape teachers' approaches to integrating digital citizenship into curricula. Finally, the study focused solely on the perspectives of teachers, overlooking the insights and experiences of students. Including student perspectives could have provided a more comprehensive understanding of the effectiveness and impact of digital citizenship education.

## 10 Recommendations for future research

Based on the responses of participants’ teachers and the discussion of the present study, the following recommendations can be made.

First, targeted training programs and professional development opportunities for teachers should be provided to enhance their digital literacy skills and proficiency in utilizing digital tools effectively in the classroom. These training programs should cater to teachers of all age groups and experience levels, recognizing the varying needs and knowledge gaps. Younger teachers may benefit from advanced training focused on innovative pedagogical strategies and the latest digital trends, while older teachers might need foundational digital skills training and integration techniques.

Secondly, the integration of digital citizenship concepts across different subject areas should be promoted to ensure a comprehensive understanding among both teachers and students. This can be achieved through collaborative efforts between subject teachers and digital citizenship specialists to develop interdisciplinary activities and projects that address various aspects of digital citizenship. Science and math classes, for example, can focus on data literacy and analysis, while social studies can explore digital democracy and ethical implications of technology.

Thirdly, it is important to recognize the grade-specific nature of digital citizenship education and provide resources, guidelines, and lesson plans that focus on addressing the unique needs, concerns, and challenges faced by students at different grade levels. For younger students, the emphasis might be on digital etiquette and online safety, while older students can delve into topics like privacy, cyberbullying, and media literacy.

Similarly, specific attention should be employed in the development of digital communication skills and fostering an understanding of digital commerce among teachers and students. This includes teaching online etiquette, safe online interactions, responsible online shopping, and skills necessary for engaging in e-commerce securely and ethically. These skills are essential for students to navigate the digital economy and make informed decisions online.

Following implementation, a balanced approach to technology use that considers the physical and mental health and well-being of students should be promoted. This could entail educating teachers and students about the potential physical effects of excessive screen time, the importance of time management, and the need to balance technology use with offline activities and social interactions. This approach helps mitigate the negative impacts of technology on students' health.

Lastly, it is important to address legal and ethical aspects of digital citizenship, such as piracy, fraud, password security, and digital rights. Equip teachers with the knowledge and resources to educate students about their rights and responsibilities in the digital world, guiding them to make informed and ethical choices while using technology. This education is crucial for developing responsible and informed digital citizens.

## 11 Conclusion

This study investigated the degree to which the elements of digital citizenship are included in the content of elementary curricula by analyzing self-reported data from teachers. Through the analysis of survey data from 300 teachers, several significant findings emerged. For instance, the analysis of grade levels revealed significant differences in mean scores for digital rights and digital health and wellness. For instance, teachers instructing grades 3, 5, and 6 placed a higher emphasis on digital rights. This phenomenon may be attributed to the more advanced developmental stage of students in these grades and, in turn, their increasing ability to comprehend and engage with concepts related to digital rights and responsibilities.

These findings highlight the need for targeted professional development programs and curriculum frameworks that consider the specific needs and perspectives of teachers at different experience levels and grade levels. Understanding the variations in teachers' perceptions and priorities regarding digital citizenship elements is crucial for designing effective educational interventions.

## Funding statement

This work was supported by 10.13039/501100004686Deanship of Scientific Research, King Faisal University, Saudi Arabia under grant number (KFU241377)

## Data availability statement

The data that has been used is confidential.

## Additional information

No additional information is available for this paper.

## CRediT authorship contribution statement

**Ashwag A. Almethen:** Writing – review & editing, Writing – original draft, Visualization, Validation, Supervision, Software, Resources, Project administration, Methodology, Investigation, Funding acquisition, Formal analysis, Data curation, Conceptualization. **Maryam A. Alomair:** Writing – review & editing, Writing – original draft, Visualization, Validation, Supervision, Software, Resources, Project administration, Methodology, Investigation, Funding acquisition, Formal analysis, Data curation, Conceptualization.

## Declaration of competing interest

The authors declare that they have no known competing financial interests or personal relationships that could have appeared to influence the work reported in this paper.
