# Supplementary material for: The role of age, grade level, and subject area in determining the inclusion of digital citizenship elements in elementary school curricula: Perspectives of teachers in the Kingdom of Saudi Arabia
Source: Heliyon. 2024 Jul 14;10(14):e34597. doi: 10.1016/j.heliyon.2024.e34597 (PMC11305296; doi:10.1016/j.heliyon.2024.e34597)
Supplement: Multimedia component 1 [file mmc1.docx]

**Questionnaire**

| item number | Items | SD | D | N | A | SA | Abbreviation |
| --- | --- | --- | --- | --- | --- | --- | --- |
| The first element: self-respect / respect for others | | | | | | |  |
| Digital Fitness and Standards of Conduct | | | | | | |  |
| 1 | The content of the curriculum develops awareness among learners of different forms of behavior It is not acceptable in digital societies (such as impersonation, spreading rumors, inappropriate images and phrases, etc.) |  |  |  |  |  | R1 |
| 2 | It helps content curriculum in identification educated how Confront for any behavior not acceptable Religiously And morally may be exposed for him via communities digital. |  |  |  |  |  | R2 |
| 3 | The content of the curriculum develops among learners the concern to respect the privacy of others in the digital world. |  |  |  |  |  | R3 |
| 4 | Curriculum content develops learners' self-monitoring when browsing websites. |  |  |  |  |  | R4 |
| 5 | The content of the curriculum contributes to presenting a culture of logical dialogue across digital societies and discussing opinions with complete objectivity for learners. |  |  |  |  |  | R5 |
| digital access | | | | | | |  |
| 6 | The content of the curriculum contributes to introducing learners to digital technology tools and its various alternatives. |  |  |  |  |  | R6 |
| 7 | The content of the curriculum encourages learners to share useful knowledge with the digital community in safe ways. |  |  |  |  |  | R7 |
| 8 | The content of the curriculum provides equal opportunities for all learners to access various digital technologies. |  |  |  |  |  | R8 |
| 9 | Curriculum content provides technical content that is appropriate and relevant to the course for learners. |  |  |  |  |  | R9 |
| 10 | Curriculum content provides high quality digital resources to learners. |  |  |  |  |  | R10 |
| digital laws | | | | | | |  |
| 11 | The content of the curriculum helps develop awareness among learners of the types of crimes in the digital society. |  |  |  |  |  | R11 |
| 12 | The content of the curriculum contributes to developing awareness among learners of the seriousness of crimes across the digital society. |  |  |  |  |  | R12 |
| 13 | The content of the curriculum informs the learners of the steps and procedures for reporting any illegal act in the digital communities. |  |  |  |  |  | R13 |
| 14 | Curriculum content encourages learners of the importance of adhering to the laws of the digital society. |  |  |  |  |  | R14 |
| 15 | The content of the curriculum develops among the learners caution against penetrating the protection programs of individuals or institutions. |  |  |  |  |  | R15 |
| The second element: self-education / communication with others | | | | | | |  |
| Digital communications | | | | | | |  |
| 16 | The content of the curriculum contributes to introducing learners to various digital communication techniques. |  |  |  |  |  | S1 |
| 17 | Curriculum content helps develop digital communication skills between learners and the community. |  |  |  |  |  | S2 |
| 18 | The content of the curriculum spreads awareness among learners of the importance of defining basic goals for communicating with others. |  |  |  |  |  | S3 |
| 19 | The content of the curriculum warns learners against accepting invitations to communicate with suspicious persons and entities. |  |  |  |  |  | S4 |
| 20 | The content of the curriculum develops among learners the culture of choosing the appropriate contact time when they want to talk to others. |  |  |  |  |  | S5 |
| Digital literacy | | | | | | |  |
| 21 | The content of the curriculum develops among learners keenness on erasing electronic digital illiteracy appropriate to scientific and research interests. |  |  |  |  |  | S6 |
| 22 | The content of the curriculum encourages learners to self-learning and continuous learning by employing digital technology. |  |  |  |  |  | S7 |
| 23 | The content of the curriculum educates learners on the latest developments in information and communication technology. |  |  |  |  |  | S8 |
| 24 | Curriculum content directs learners to learn how to make use of technology in educational, social, or research aspects. |  |  |  |  |  | S9 |
| 25 | The content of the curriculum helps learners to practically train them on the skills of contemporary digital technology. |  |  |  |  |  | S10 |
| digital trade | | | | | | |  |
| 26 | The content of the curriculum contributes to developing learners' awareness of how to select the best e-commerce websites. |  |  |  |  |  | S11 |
| 27 | The content of the curriculum teaches learners how to read the certificate of credibility of any online shopping website. |  |  |  |  |  | S12 |
| 28 | The content of the curriculum directs learners towards rationalizing and legalizing electronic shopping operations. |  |  |  |  |  | S13 |
| 29 | The content of the curriculum develops awareness among learners of the importance of avoiding buying and selling products and services that are inconsistent with the laws and regulations of their country. |  |  |  |  |  | S14 |
| 30 | The curriculum content develops learners' awareness of the importance of correct dealing with annoying commercial messages on learners' devices. |  |  |  |  |  | S15 |
| The third element: self-protection/protection of others | | | | | | |  |
| Digital rights and responsibilities | | | | | | |  |
| 31 | Curriculum content informs learners of their rights and responsibilities in the digital world. |  |  |  |  |  | P1 |
| 32 | The content of the curriculum highlights the importance of reading the policy of any website before registering with it or dealing with it for learners. |  |  |  |  |  | P2 |
| 3 3 | The content of the curriculum educates learners on ways to deal with any misinformation against government policy through social networks. |  |  |  |  |  | P3 |
| 34 | The content of the curriculum warns learners against contributing to spreading rumors through digital communities. |  |  |  |  |  | P4 |
| 35 | The content of the curriculum highlights the role of learners in spreading peace, tolerance, and renunciation of violence and aggression in the digital world. |  |  |  |  |  | P5 |
| Digital security | | | | | | |  |
| 36 | The content of the curriculum contributes to educating learners about methods and programs that ensure prevention, protection, and safety in the digital society. |  |  |  |  |  | P6 |
| 37 | The content of the curriculum develops awareness among learners of the skills and how to choose passwords that are difficult to crack. |  |  |  |  |  | P7 |
| 38 | The content of the curriculum develops awareness among learners of piracy and fraud. |  |  |  |  |  | P8 |
| 39 | Curriculum content provides learners with suggestions for safe Internet browsers. |  |  |  |  |  | P9 |
| 40 | The content of the curriculum develops awareness among learners of cyberbullying and ways to prevent it. |  |  |  |  |  | P10 |
| Digital health and safety | | | | | | |  |
| 41 | The content of the curriculum informs learners of the risks inherent in technology related to physical and psychological health and safety. |  |  |  |  |  | P11 |
| 42 | The content of the curriculum develops awareness among learners of the physical effects of using electronic devices for long periods. |  |  |  |  |  | P12 |
| 43 | Curriculum content focuses on demonstrating the importance of time management when using technology to avoid risks of addiction among learners. |  |  |  |  |  | P13 |
| 44 | The content of the curriculum educates learners about the psychological damage that may result from integrating into digital societies. |  |  |  |  |  | P14 |
| 45 | The content of the curriculum contributes to teaching learners to balance the positive and negative aspects of mental and physical health when using technology. |  |  |  |  |  | P15 |
